# The Prognostic Performance of Artificial Intelligence and Machine Learning Models for Mortality Prediction in Intensive Care Units: A Systematic Review

**DOI:** 10.7759/cureus.90465

**Published:** 2025-08-19

**Authors:** Archana Dhami, Kosisochukwu A Onyeukwu, Saba Sattar, Anmol Batra, Yahia Mostafa, Muhammad Haris, Asma Iqbal, Syed Faqeer Hussain Bokhari, Muhammad Uzair Siddique

**Affiliations:** 1 Family Medicine, Avalon University School of Medicine, Willemstad, CUW; 2 General Practice, National Health Service (NHS), Gateshead, GBR; 3 Internal Medicine, King Edward Medical University, Lahore, PAK; 4 Internal Medicine, Ghulam Muhammad Mahar Medical College, Sukkur, PAK; 5 Medicine, Ain Shams University, Cairo, EGY; 6 Medicine and Surgery, King Edward Medical University, Lahore, PAK

**Keywords:** artificial intelligence, ccu, critical care, icu, intensive care, machine learning, mortality, mortality prediction

## Abstract

In-hospital mortality prediction for patients admitted to the ICU remains a critical challenge in the field of critical care medicine. This systematic review evaluates the application of artificial intelligence (AI) and machine learning (ML) models for predicting in-hospital mortality in ICU settings. Following the Preferred Reporting Items for Systematic Reviews and Meta-Analyses (PRISMA) guidelines, we analyzed 15 studies published between January 2015 and April 2025 that utilized AI and ML approaches for mortality prediction in ICU populations. The most commonly employed algorithms were extreme gradient boosting (XGBoost), random forest, and logistic regression, with data predominantly sourced from two major publicly available critical care databases: the Medical Information Mart for Intensive Care (MIMIC) and the eICU Collaborative Research Database (eICU-CRD).

Across all studies, AI and ML models consistently outperformed traditional clinical scoring systems such as the Acute Physiology and Chronic Health Evaluation (APACHE), Sequential Organ Failure Assessment (SOFA), and Simplified Acute Physiology Score (SAPS), demonstrating superior discriminative performance in mortality prediction. Ensemble methods, particularly random forest and XGBoost, generally achieved the highest predictive accuracy, while deep learning approaches such as recurrent neural networks showed particular promise for analyzing temporal trends in physiological data. Key predictive features identified across multiple studies included patient age, vital signs (especially heart rate and blood pressure), laboratory values (particularly markers of renal function), and metrics of neurological status such as level of consciousness. Importantly, several studies developed models that required only routinely collected clinical data available within the first 24 hours of ICU admission, demonstrating the feasibility of early risk stratification using AI and ML.

Although most research remains retrospective in nature and confined to a limited number of datasets, the consistent performance advantages observed across diverse modeling approaches underscore the significant clinical potential of AI and ML in ICU mortality prediction. Future research should prioritize the use of standardized model development and reporting methodologies, prospective validation in diverse and real-world clinical settings, exploration of integration and implementation challenges, and rigorous assessments of clinical impact. Such efforts are critical to translating these promising predictive technologies into improved decision-making processes and outcomes for critically ill patients.

## Introduction and background

The ICU plays a critical role in managing life-threatening conditions, offering close monitoring and advanced medical interventions for patients with severe illnesses or injuries. Despite the technological advancements and standardized treatment protocols in modern ICUs, in-hospital mortality rates remain high. Predicting mortality accurately in ICU patients is crucial for informed clinical decision-making, optimal resource allocation, and early intervention strategies [1]. Traditional scoring systems such as APACHE (Acute Physiology and Chronic Health Evaluation), SOFA (Sequential Organ Failure Assessment), and SAPS (Simplified Acute Physiology Score) have been widely used to estimate mortality risk [2,3]. However, these models are often limited by their reliance on predefined variables, linear relationships, and static measurements, which may not fully capture the complex, dynamic physiology of critically ill patients.

In recent years, artificial intelligence (AI) and machine learning (ML) have emerged as transformative tools in healthcare, particularly in the realm of predictive analytics. Unlike traditional statistical methods, AI and ML techniques can analyze large volumes of heterogeneous data, detect non-linear patterns, and continuously learn from new inputs to enhance predictive performance. This capability makes them particularly suitable for modeling the multifactorial nature of patient outcomes in the ICU, where data are high-dimensional, time-sensitive, and frequently updated [4].

Several studies have explored the application of AI and ML in predicting mortality among ICU patients, leveraging electronic health records (EHRs), physiological signals, laboratory results, imaging, and other clinical data. Algorithms such as decision trees, random forests, support vector machines (SVM), gradient boosting machines (GBM), and deep learning neural networks have shown promising results in various clinical settings. Furthermore, ensemble methods and hybrid models that combine multiple algorithms have demonstrated superior performance in certain cases. These models aim not only to improve predictive accuracy but also to enable real-time risk stratification, thereby enhancing patient safety and operational efficiency in critical care [5].

Despite the growing body of literature, the clinical translation of AI and ML-based prediction models remains limited. A key barrier involves the lack of standardization in study design, data preprocessing, feature selection, validation techniques, and outcome measures. Concerns around model interpretability, generalizability across populations, and ethical implications, such as bias and accountability, pose further challenges. There is also a gap in understanding how these models perform in comparison to traditional scoring systems and whether their integration into clinical workflows can have a meaningful impact on patient outcomes. Given the proliferation of AI/ML studies in this area, there is a pressing need to systematically evaluate their methodological quality, performance metrics, and clinical applicability. Previous reviews have often adopted a narrative design or focused on specific algorithms or datasets, lacking comprehensive synthesis. A systematic review can provide a more holistic understanding by aggregating evidence, identifying trends, highlighting limitations, and guiding future research directions.

This systematic review aims to evaluate the safety, efficacy, and clinical performance of AI and ML models for predicting in-hospital mortality in ICU patients. Specifically, we will examine (1) the types of AI/ML algorithms used, (2) the nature and quality of data inputs, (3) the outcome measures and validation methods employed, and (4) the comparative effectiveness of these models against traditional clinical scoring systems. By synthesizing current evidence, this review will provide clinicians, data scientists, and healthcare policymakers with critical insights into the practical utility of AI and ML in critical care settings. It also aims to identify research gaps, methodological weaknesses, and areas for future investigation. Ultimately, our goal is to facilitate the responsible and evidence-based integration of AI into ICU care, with the hope of improving patient outcomes and system efficiency.

## Review

Materials and methods

This systematic review was conducted in accordance with the Preferred Reporting Items for Systematic Reviews and Meta-Analyses (PRISMA) 2020 guidelines [6].

Search Strategy

A comprehensive literature search was conducted across five electronic databases: PubMed, Scopus, Hinari, and the Cochrane Library. In addition to database searching, the reference lists of included articles and relevant review papers were manually screened to identify any additional eligible studies. The search strategy incorporated both Medical Subject Headings (MeSH) and free-text keywords, including terms such as "artificial intelligence," "machine learning," "deep learning," "predictive modeling," "mortality," "ICU," "critical care," and "in-hospital." Searches were limited to articles published from January 1, 2015, to April 5, 2025, in English.

Eligibility Criteria

Eligible studies were included based on predefined inclusion and exclusion criteria. Studies were considered eligible if they applied AI or ML algorithms to predict in-hospital mortality in ICU settings, involved human participants, and reported performance metrics such as accuracy, area under the curve (AUC), sensitivity, specificity, or precision-recall. Only original, peer-reviewed research articles published in English from January 2015 to April 2025 were included. Studies were excluded if they focused on non-ICU patient populations, outcomes other than in-hospital mortality (such as 30-day or long-term mortality), or employed conventional statistical models without AI or ML methods. Editorials, commentaries, reviews, meta-analyses, conference abstracts without full texts, and animal studies were also excluded.

Study Selection

All retrieved articles were imported into the Rayyan software platform for systematic screening. Two independent reviewers screened titles and abstracts to identify potentially eligible studies. The full texts of articles deemed relevant were assessed in detail using the inclusion and exclusion criteria. Disagreements during the selection process were resolved through discussion or consultation with a third reviewer when necessary.

Data Extraction

Data from each included study were extracted using a standardized and pilot-tested data extraction form. The data collected included study characteristics (such as author, year of publication, country, and study design), population details (sample size, ICU type), types of AI or ML models used, data input features (clinical variables, laboratory results, imaging, etc.), outcome definition (in-hospital mortality), and performance metrics (AUC, accuracy, sensitivity, specificity, etc.). Two reviewers independently performed the data extraction, and any discrepancies were resolved through consensus.

Data Synthesis

A narrative synthesis was conducted due to the anticipated heterogeneity in model types, performance metrics, and data sources. Study characteristics, AI/ML methods, and predictive performances were summarized in tabular form. Quantitative synthesis (meta-analysis) was not performed due to methodological variability. Trends, strengths, and limitations were discussed qualitatively.

Results

Study Selection Process

**Figure 1:**
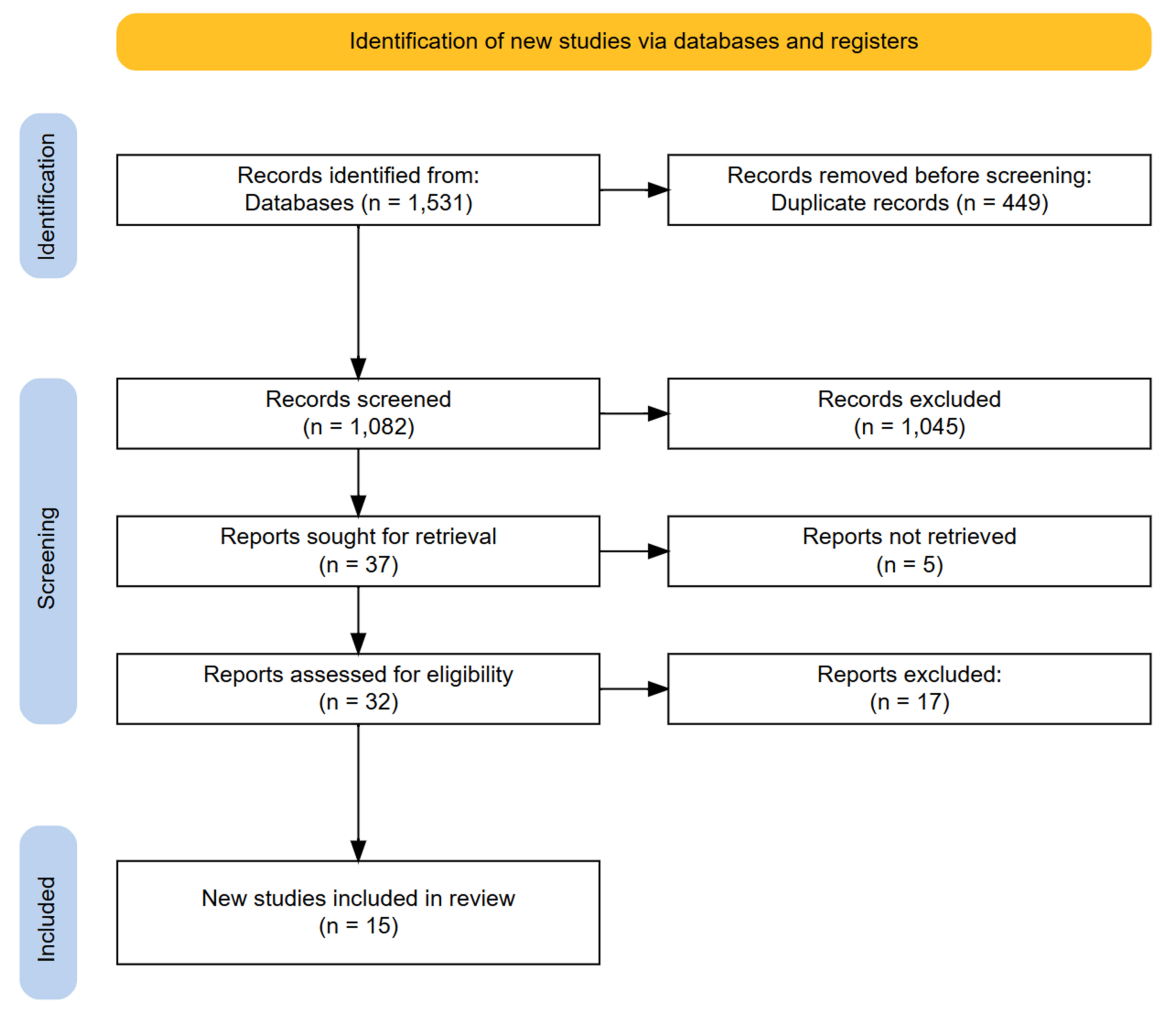
PRISMA diagram illustrating the study selection process PRISMA: Preferred Reporting Items for Systematic Reviews and Meta-Analyses

A total of 1531 records were retrieved through the initial database searches. After removing 449 duplicates, 1082 articles remained for title and abstract screening. Following this step, 37 full-text articles were assessed for eligibility. Ultimately, 15 studies met the inclusion criteria and were included in the final review. The PRISMA flow diagram illustrating the study selection process is provided in the following figure (Figure 1).

Study Characteristics

All 15 studies included in the review utilized retrospective cohort designs. The primary data sources were MIMIC databases (MIMIC-III appeared in nine studies and MIMIC-IV in three studies), followed by eICU-CRD (four studies). Some studies used institution-specific data sources, such as the YCC and SMC databases in Ko et al. (2023) or the Danish EPRs in Thorsen-Meyer et al. (2020) [7,8]. Sample sizes varied widely, from 336 patients in Hu et al. (2020) to over 44,000 in Alghatani et al. (2021). Publication dates ranged from 2020 to 2025, with most published between 2020 and 2023. The predominant outcome was in-hospital mortality, though some studies examined specific timeframes: 28-day mortality (Huang et al., 2023; Ko et al., 2023), 30-day mortality (Hu et al., 2020), and 90-day mortality (Thorsen-Meyer et al., 2020) [7,9,10]. Various ML algorithms were employed across studies, with XGBoost appearing in eight studies, random forest in seven studies, and logistic regression in seven studies. Neural network approaches, including LSTM architectures, were used in three studies. All studies compared their ML models against traditional clinical scoring systems like APACHE, SAPS, or SOFA, consistently demonstrating superior performance of the AI/ML approaches (Table 1).

**Table 1:** Summary of the main findings of the included studies LR: logistic regression; RF: random forest; SVM: support vector machine; XGBoost: extreme gradient boosting; GBM: gradient boosting machine; LASSO: least absolute shrinkage and selection operator; kNN: k-nearest neighbors; LDA: linear discriminant analysis; CNN: convolutional neural network; BiLSTM: bidirectional long short-term memory; ANN: artificial neural network; APACHE: Acute Physiology and Chronic Health Evaluation; SOFA: Sequential Organ Failure Assessment; SAPS: Simplified Acute Physiology Score; PSI: Pneumonia Severity Index; APS: Acute Physiology Score; LODS: Logistic Organ Dysfunction System; NEWS2: National Early Warning Score 2; GWTG-HF: Get With the Guidelines-Heart Failure; ICU: intensive care unit; AUROC/AUC: area under the receiver operating characteristic curve; BUN: blood urea nitrogen; GCS: Glasgow Coma Scale; SpO_2_: oxygen saturation); BP: blood pressure; ALT: alanine aminotransferase; AST: aspartate aminotransferase; WBC: white blood cell; MCV: mean corpuscular volume; RDW: red cell distribution width; PT: prothrombin time; Hb: hemoglobin; AD: aortic dissection; SBP: systolic blood pressure; DBP: diastolic blood pressure; MAP: mean arterial pressure; AIECG: artificial intelligence electrocardiogram; LVDD: left ventricular diastolic dysfunction; TTE: transthoracic echocardiography

Author	Year	Study design	Data source	Sample size	Outcomes studied	Models studies	Risk factors identified	Main findings	Conclusions
Baker et al. [11]	2020	Retrospective cohort	MIMIC-III	51,639 adult ICU patients (for AIMS-14 model; slight variations for other models)	Mortality within 3-day, 7-day, and 14-day windows	Hybrid neural network approach combining CNN layers with BiLSTM	Variations in vital signs (heart rate, blood pressure, respiratory rate, blood oxygen levels, temperature), age, and gender	Best performing model (AIMS-3) achieved an AUROC of 0.884. The AIMS-7 and AIMS-14 models achieved AUROCs of 0.862 and 0.858, respectively	The use of a hybrid CNN-BiLSTM network is highly effective in determining mortality risk for 3-, 7-, and 14-day windows from vital signs. As vital signs are routinely recorded, in many cases automatically, their scheme could be implemented such that highly accurate mortality risk could be predicted continuously and automatically, reducing the burden on healthcare providers and improving patient outcomes.
Hu et al. [10]	2020	Cross-sectional study	Eight medical centers in Taiwan	336 critically ill influenza patients requiring ICU admission	30-day mortality	XGBoost, RF, LR	Fluid balance domain (0.253 importance), severity score domain (0.165), laboratory data domain (0.177), management domain (0.152), demographic and symptom domain (0.140), ventilation domain (0.113). Key individual factors: PSI score, cumulative day-4 fluid balance, PaO_2_/FiO_2_ ratio	XGBoost model (AUC: 0.842) outperformed RF (AUC: 0.809) and LR (AUC: 0.701) for predicting 30-day mortality. Both APACHE II and PSI had an AUC of 0.720	The ML approach, particularly XGBoost, established a practical and explainable mortality prediction model with higher accuracy than traditional severity scoring systems (APACHE II and PSI)
Kong et al. [10]	2020	Retrospective cohort	MIMIC-III	16,688 sepsis patients	In-hospital mortality	LASSO, RF, GBM, LR, SAPS II	86 predictor variables consisting of demographics, laboratory tests, and comorbidities, including vital signs, SOFA score, age, gender, and multiple other clinical parameters measured in the first 24 hours of ICU admission	The GBM model showed the best performance with an AUROC of 0.845 and a Brier score of 0.104. All ML models (LASSO: AUROC 0.829, RF: AUROC 0.829, LR: AUROC 0.833) outperformed SAPS II (AUROC 0.77). GBM, LASSO, and LR models had good calibration, while the RF model underestimated high-risk patients	ML-based models, especially GBM, performed well in predicting in-hospital mortality of sepsis patients. The GBM model showed the best performance and has the potential to assist physicians in the ICU to perform appropriate clinical interventions for critically ill sepsis patients, possibly improving prognoses
Yu et al. [12]	2020	Retrospective cohort	MIMIC-III	22,049 unique in-hospital admissions (89.5% discharged, 10.5% died)	In-hospital mortality	LSA embeddings with bidirectional LSTM, unidirectional LSTM, self-attention mechanism, and average pooling models compared against SAPS-II	Medical events, including laboratory tests, vital signs, and medications	Bidirectional LSTM demonstrated superior performance (AUROC 0.8854, AUPRC 0.3184) compared to other models and the traditional SAPS-II scoring system (AUROC 0.7749, AUPRC 0.1051). The model captures clinically meaningful representations that correlate with SAPS-II scores despite not being exposed to the explicit values used in SAPS-II calculation	The approach of using data-driven feature representations combined with RNN methods consistently outperforms traditional SAPS-II features. The model can be used as an instant scoring system to help ICU clinicians assess patient severity. The bidirectional LSTM performs best by capturing both forward and backward temporal dependencies in patient data
Thorsen-Meyer et al. [7]	2020	Retrospective cohort	EPRs from 4 mixed medical and surgical ICUs in Denmark	14,190 ICU admissions (11,492 patients) for development; 6,748 ICU admissions (5,827 patients) for external validation	90-day mortality after ICU admission	Recurrent neural network with LSTM architecture	Age was the most important feature; other significant features included heart frequency, systolic blood pressure, Glasgow Coma Scale, mechanical ventilation, and various laboratory values	Model performance improved throughout ICU stay: AUROC of 0.73 at admission, 0.82 after 24 hours, 0.85 after 72 hours, and 0.88 at discharge. External validation showed a similar pattern with slightly lower performance. Features can drive prediction toward survival at one time point and toward non-survival at another	Dynamic, explainable ML models for mortality prediction can outperform static scores by updating predictions hourly, incorporating temporal developments in patient status. The explainability of the model allows clinicians to identify which factors are currently influencing mortality risk, making it suitable for further validation as a clinical tool
Alghatani et al. [13]	2021	Retrospective cohort	MIMIC-III	44,626	In-hospital mortality, ICU length of stay (LOS)	LR, LDA, RF, kNN, SVM, XGB	Vital signs (heart rate, systolic and diastolic BP, respiration rate, temperature, SpO_2_, glucose) demographics (age, gender, height, weight)	RF (quantiles) achieved the highest mortality prediction accuracy (~88.61%), while XGB had the highest AUROC (0.79). Vital signs + demographics with engineered features (quantiles) yielded reasonable performance. Quantiles approach modestly improved mortality prediction and significantly improved LOS prediction	ML models can predict ICU mortality and LOS with reasonable accuracy using only routinely collected vital signs and demographics. The quantiles approach improves feature richness and predictive power without requiring complex clinical data
Li et al. [14]	2021	Retrospective cohort	MIMIC-III	1,177 ICU patients with heart failure (825 in the derivation cohort, 352 in the validation cohort)	In-hospital mortality	XGBoost algorithm, LASSO regression, and multivariate logistic regression (compared with the GWTG-HF risk score model)	Six key variables: anion gap, lactate levels, calcium levels, BUN, CKD status, and diastolic blood pressure	In-hospital mortality was 13.52% for ICU patients with heart failure. XGBoost model achieved AUC of 0.8416 (95% CI 0.7864-0.8967). XGBoost and LASSO models outperformed the existing GWTG-HF risk score model. Non-linear relationships were found between mortality risk and both anion gap (>14.73 mEq/L) and lactate level (>1.5 mmol/L)	The nomogram enabled good prediction of in-hospital mortality in ICU-admitted heart failure patients, which may help clinical decision-making. The model had a high AUC of 0.8416 and a wide net benefit threshold range (>0.1), using only a small number of routinely collected variables that can be easily applied at bedside
Li et al. [15]	2022	Retrospective cohort	eICU-CRD	2,798 patients with heart failure	In-hospital mortality	XGBoost, LR, RF, SVM	Top 5: BUN, age, noninvasive systolic blood pressure, average urine output, maximum respiratory rate	The XGBoost model had the highest predictive performance (AUC=0.824, 95% CI 0.7766-0.8708). SVM had the poorest performance (AUC=0.701). The net benefit of XGBoost surpassed other models at 10%~28% threshold probabilities	The interpretable XGBoost prediction model has the best performance in estimating mortality risk in HF patients. The interpretable ML approach can accurately explore risk factors and enhance trust in prediction models, helping identify HF patients with high mortality risk for timely treatments
Pang et al. [16]	2022	Retrospective cohort	MIMIC-IV	14,110 patients (7,055 deceased and 7,055 surviving patients randomly selected through downsampling)	In-hospital mortality	XGBoost, LR, SVM, Decision Tree	GCS score, respiratory rate score, acid base score, urine output score, age, weight, APS III total score	The XGBoost model achieved the highest performance with an AUC of 0.918. Logistic regression and SVM both achieved an AUC of 0.872. The decision tree achieved an AUC of 0.852. XGBoost showed better accuracy (0.834), sensitivity (0.822), and specificity (0.846) compared to other models. Calibration curves showed that logistic regression and SVM performed better for patients with low (0-40%) and high (70-100%) mortality risk, while XGBoost performed better for patients with medium mortality risk (40-70%)	The mortality risk of ICU patients can be better predicted using the APS III and LODS with the XGBoost algorithm in terms of ROC curve, sensitivity, and specificity. The XGBoost model could assist clinicians in judging the in-hospital outcome of critically ill patients, especially in patients with more uncertain survival outcomes
Safaei et al. [17]	2022	Predictive modeling study with 10-fold cross-validation	eICU-CRD	>200,000 ICU admissions across 208 U.S. hospitals (2014-2015)	ICU mortality prediction upon discharge	CatBoost and E-CatBoost were compared against other ML models such as XGBoost, LightGBM, and RF, as well as scoring systems (APACHE IVa, SAPS II, and SOFA)	Age, heart rate, respiratory rate, BUN, blood creatinine level, and verbal score were identified as the most important cross-disease features	CatBoost AUROC: 0.91 (std: 0.0038) across entire population, 0.86-0.92 across disease groups; E-CatBoost AUROC: 0.87 (std: 0.004) across entire population, 0.83-0.91 across disease groups; both models outperformed traditional severity scoring systems by 2-18%	The proposed E-CatBoost model provides accurate mortality predictions using only 10 input features collected during the first 24 hours of ICU admission, making it more efficient than traditional scoring systems while maintaining high performance. The model also offers explainability through SHAP, LIME, and other visualization techniques to identify key risk factors and their critical ranges
Huang et al. [9]	2023	Retrospective cohort	MIMIC-IV and eICU-CRD	4,274 hypertensive ischemic or hemorrhagic stroke patients admitted to the ICU	28-day all-cause in-hospital mortality	ANN, GBM, XGBoost, LR, SVM	Age, ethnicity, WBC, hyperlipidemia, MCV, glucose, SpO₂, serum calcium, RDW, BUN, bicarbonate	The XGBoost model demonstrated the best predictive performance with AUC values of 0.822, 0.739, and 0.700 in the training, test, and external cohorts, respectively. Feature importance analysis revealed that demographic characteristics (age, ethnicity), laboratory tests (glucose, WBC, calcium, BUN, MCV, RDW, bicarbonate), vital signs (SpO₂), and comorbidity (hyperlipidemia) significantly affected the predictive models. The SHAP method was used to provide interpretability of the ML model	The XGBoost model accurately predicted 28-day all-cause in-hospital mortality. The SHAP method provided explicit explanations of personalized risk prediction, which can aid physicians in understanding the model. The identified cutoff values for features including SpO₂ (98%), age (71.27 years), MCV (91 fl), RDW (13.6%), bicarbonate (24 mmol/L), BUN (17 mg/dL), calcium (8.8 mg/dL), and glucose (130 mg/dL) can be used to identify high-risk patients
Ko et al. [8]	2023	Retrospective cohort	YCC (Yonsei Cancer Center) from 2008 to 2017, SMC (Samsung Medical Center) from 2011 to 2017, and the MIMIC-III database from 2001 to 2012	Total: 6,900 patients (YCC: 3,571; SMC: 2,563; MIMIC-III: 766)	28-day and 1-year mortality	RF (CanICU), compared with SOFA score and APACHE-III score; other models tested included XGBoost and SVM	Nine variables: primary reason for ICU admission (medical vs. surgical), BUN, heart rate, PaO₂/FiO₂ ratio, pH, albumin, lactate, PT, and Hb	CanICU showed better performance than conventional scores (SOFA and APACHE-III) with high sensitivity (95.5%/88.9%/78.6% in YCC/SMC/MIMIC-III) and high NPV (99.3%/97.3%/82.7%). AUC for 28-day mortality: 0.939 in YCC, 0.775 in SMC, 0.753 in MIMIC-III. 28-day mortality rates: 10.2%/12.7%/36.6% in YCC/SMC/MIMIC-III cohorts	CanICU offers improved performance for predicting short-term and long-term mortality in critically ill cancer patients admitted to the ICU. The model uses nine variables that can be easily obtained in practical ICU settings and can help physicians determine how to allocate ICU care for cancer patients according to objective mortality risk
Lei et al. [18]	2023	Retrospective cohort	MIMIC-IV and eICU-CRD	Patients with AD. 643 ICU admissions from MIMIC-IV and 501 from eICU	In-hospital mortality	Multivariable LR, Simple Decision Tree, RF, and XGBoost	Mean 24-h fluid intake, baseline blood pressure, mean blood pressure, creatinine, ALT, AST, heart failure, congenital disorders, aortic rupture	RF models for SBP/DBP and MAP subsets showed the best performance with AUCs of 0.870 and 0.850. Mean 24-h fluid intake was consistently the most important risk factor	The developed prognostic models effectively forecasted in-hospital mortality among ICU patients with AD. Notable prognostic factors included initial blood pressure upon ICU admission and mean 24-h fluid intake
Sun et al. [19]	2023	Retrospective cohort	MIMIC-IV	1,722 ICU patients with cardiac arrest	In-hospital mortality	LASSO regression, XGBoost algorithm, LR, NEWS2	Age, SAPS III score, heart rate, mean blood pressure, respiratory rate, temperature, SPO₂, GCS score, gender (male), bicarbonate level, prothrombin time	In-hospital mortality was 53.95% for cardiac arrest patients. LASSO model showed best performance with AUC of 0.7912 (95% CI 0.7703-0.8122). Prediction models (LASSO, XGBoost, LR) significantly outperformed the NEWS2 scoring system. Variables like hypertension, diabetes, and vasopressor use were not significant predictors	The LASSO model enables good prediction of in-hospital mortality in ICU cardiac arrest patients and may be widely applicable for clinical decision-making
Jentzer et al. [20]	2025	Retrospective cohort	Mayo Clinic cardiac ICU database (January 2007 to April 2018)	11,868 unique adult patients	In-hospital mortality and 1-year mortality	AIECG algorithm for LVDD	Higher AIECG LVDD grade, higher probability of elevated filling pressures, concordant AIECG and TTE for elevated filling pressures	AIECG LVDD grade was strongly associated with in-hospital mortality (adjusted OR 1.22 [95% CI, 1.13-1.32] per grade) and 1-year mortality (adjusted HR 1.23 [95% CI, 1.19-1.29] per grade). Patients with grade 2 or 3 LVDD by AIECG and medial mitral E/e' ratio >15 by TTE had the highest mortality	AIECG algorithm for LVDD strongly predicted short-term and intermediate mortality in cardiac ICU patients, even after adjusting for clinical variables and TTE measurements. The AIECG prediction of LVDD was superior to the medial mitral E/e' ratio for prognostication. AIECG can serve as both a diagnostic and prognostic tool in addition to TTE assessment in cardiac ICU patients

Discussion

The findings of this systematic review highlight the significant potential of AI and ML approaches in predicting in-hospital mortality among ICU patients. Across all 15 included studies, AI/ML models consistently demonstrated superior predictive performance compared to traditional clinical scoring systems such as APACHE, SOFA, and SAPS. This performance advantage is evidenced by higher AUROCs, better sensitivity/specificity values, and improved calibration metrics in most cases. Several key trends emerged from our analysis. First, ensemble methods, particularly XGBoost and random forest, appeared in a majority of the studies and frequently achieved the highest predictive performance. The success of these algorithms can be attributed to their ability to capture complex, non-linear relationships between clinical variables and mortality outcomes, as well as their robustness to noise and missing data, common challenges in ICU datasets. For instance, the studies by Huang et al. (2023), Li et al. (2022), and Ko et al. (2023) all demonstrated the effectiveness of ensemble approaches in different subpopulations of critically ill patients [8,9,15].

Secondly, while deep learning approaches were less frequently employed, they showed promising results, especially for time-series data analysis. Baker et al. (2020) and Yu et al. (2020) demonstrated that neural network architectures incorporating temporal dependencies (CNN-BiLSTM and LSTM, respectively) could effectively leverage the sequential nature of vital signs and clinical events to improve mortality predictions [11,12]. Similarly, Thorsen-Meyer et al. (2020) showed how recurrent neural networks could provide continuously updated risk assessments that improved in accuracy as the ICU stay progressed, a dynamic capability that static scoring systems lack [7]. Third, the input features utilized across studies reveal important patterns. While some models incorporated hundreds of variables, several high-performing models achieved excellent results with relatively modest feature sets. Vital signs, laboratory values (particularly renal function markers like BUN and creatinine), age, and measures of consciousness (e.g., GCS) were consistently identified as important predictors across multiple studies and populations. This suggests that carefully selected, clinically relevant variables may be sufficient for accurate mortality prediction, potentially facilitating model implementation in resource-constrained settings.

The explainability of AI/ML models emerged as an important consideration. Techniques such as SHAP (Huang et al., 2023; Safaei et al., 2022) and feature importance analysis provided interpretable insights into model predictions, identifying specific thresholds for clinical variables that significantly impacted mortality risk [9,17]. For example, Huang et al. (2023) identified clinically meaningful cutoff values for SpO₂ (98%), age (71.27 years), and laboratory parameters that could help clinicians identify high-risk stroke patients [9]. This transparency addresses a critical barrier to clinical adoption of AI systems, potentially enhancing physician trust and facilitating integration into clinical decision-making. Another significant finding was the ability of AI/ML models to predict mortality in specific ICU subpopulations with high accuracy. Studies targeting patients with heart failure (Li et al., 2021, 2022), sepsis (Kong et al., 2020), stroke (Huang et al., 2023), cancer (Ko et al., 2023), aortic dissection (Lei et al., 2023), and cardiac arrest (Sun et al., 2023) all demonstrated successful application of these techniques to specialized cohorts [8,9,14,15,18,21]. This suggests that while general ICU mortality models have value, condition-specific models may offer additional precision for particular patient groups.

The generalizability of AI/ML models across different healthcare settings represents a promising yet challenging aspect of this research. Some studies, such as Ko et al. (2023), evaluated their models across multiple institutions and datasets (YCC, SMC, and MIMIC-III), finding varying but generally acceptable performance [8]. Thorsen-Meyer et al. (2020) specifically included external validation across different Danish ICUs. These approaches help address concerns about the robustness of AI/ML models when applied outside their development environments, although performance typically decreased somewhat in external validation cohorts [7].

From a practical implementation perspective, several studies demonstrated that AI/ML models could achieve high predictive accuracy using routinely collected clinical data available within the first 24 hours of ICU admission. This early predictive capability could enable timely risk stratification and intervention, potentially improving resource allocation and patient outcomes. Furthermore, studies like Baker et al. (2020) explored mortality prediction at multiple time horizons (3, 7, and 14 days), providing flexibility to match prediction windows with clinical needs [11]. Overall, this systematic review suggests that AI/ML approaches represent a significant advancement over traditional scoring systems for ICU mortality prediction. Their ability to analyze large, complex datasets, capture non-linear relationships, and provide dynamic risk assessments aligns well with the multifactorial and evolving nature of critical illness.

Limitations and future directions

Despite the promising results, this systematic review identified several limitations in the current body of research that must be addressed in future work. First, methodological heterogeneity across studies, in terms of datasets, preprocessing techniques, feature selection approaches, and evaluation metrics, makes direct comparison challenging. Standardization of reporting and methodology would facilitate more robust meta-analyses and clearer identification of superior approaches. Second, most studies relied on retrospective data from a limited number of databases, with MIMIC and eICU-CRD being predominant sources. While these are valuable resources, they may not represent the full diversity of ICU settings worldwide. Future research should incorporate data from more varied healthcare systems, particularly from resource-limited settings and different geographical regions, to enhance generalizability. Third, the clinical implementation and impact assessment of these AI/ML models remain largely unexplored. Only one study extended beyond development and validation to examine how model integration might affect clinical decision-making or patient outcomes.

Prospective, interventional studies are needed to determine whether predictive accuracy translates to clinical utility and improved patient care. Fourth, while some studies addressed model interpretability, many treated AI/ML algorithms as "black boxes," limiting their potential acceptability among clinicians. Future work should prioritize explainable AI approaches that provide transparency into the reasoning behind predictions. Fifth, ethical considerations, including algorithmic bias and fairness across demographic groups, were rarely addressed. Given documented disparities in healthcare, ensuring that AI/ML models do not perpetuate or amplify biases is crucial for equitable implementation.

Future research directions should include: (1) development of models that incorporate temporal dynamics more effectively, possibly through transfer learning or federated learning approaches; (2) integration of multimodal data sources, including imaging and unstructured clinical notes; (3) prospective validation studies in diverse clinical settings; (4) investigation of model updating strategies to maintain performance over time; and (5) comprehensive evaluation of implementation barriers from technical, organizational, and human factors perspectives.

## Conclusions

This systematic review demonstrates that AI and ML approaches consistently outperform traditional scoring systems in predicting in-hospital mortality among ICU patients. Ensemble methods like XGBoost and random forest typically achieved the highest performance, while deep learning models showed particular promise for temporal data analysis. The successful application of these techniques across various ICU subpopulations suggests broad clinical utility. However, methodological heterogeneity, limited prospective validation, and insufficient attention to implementation challenges currently restrict widespread clinical adoption. Future research addressing these limitations could transform ICU mortality prediction, enabling more personalized risk assessment, improved resource allocation, and ultimately, better patient outcomes through early intervention for those at highest risk.
